# Association of systemic lupus erythematosus standard of care immunosuppressants with glucocorticoid use and disease outcomes: a multicentre cohort study

**DOI:** 10.1186/s42358-024-00366-y

**Published:** 2024-05-08

**Authors:** Ricardo Azêdo de Luca Montes, Molla Huq, Timothy Godfrey, Shereen Oon, Alicia Calderone, Rangi Kandane-Rathnayake, Worawit Louthrenoo, Shue-Fen Luo, Yeong-Jian Jan Wu, Vera Golder, Aisha Lateef, Sandra V. Navarra, Leonid Zamora, Laniyati Hamijoyo, Sargunan Sockalingam, Yuan An, Zhanguo Li, Yasuhiro Katsumata, Masayoshi Harigai, Madelynn Chan, Fiona Goldblatt, Sean O’Neill, Chak Sing Lau, Jiacai Cho, Alberta Hoi, Chetan S. Karyekar, Eric F. Morand, Mandana Nikpour

**Affiliations:** 1 Rheumatology Department, Universidade Federal do Rio de Janeiro, Rio de Janeiro, Brazil; 2 Internal Medicine, Universidade do Estado do Rio De Janeiro, Rua São Francisco Xavier 524, Maracanã, Rio de Janeiro, 20550-900 Brazil; 3 Department of Medicine, The University of Melbourne at St Vincent’s Hospital, 41 Victoria Parade, Fitzroy, Victoria 3065 Australia; 4 Department of Rheumatology, St Vincent’s Hospital Melbourne, 41 Victoria Parade, Fitzroy, Victoria 3065 Australia; 5 Monash University, Level 5, Block E, Monash Medical Centre, 246 Clayton Road, Clayton, Victoria 3168 Australia; 6 Chiang Mai University Hospital, 110 Intravororos Street, Muang District, Chang Mai, 50200 Thailand; 7 Chang Gung Memorial Hospital, 5 Fuxing Street, Guishan Township, Taoyuan County, 333 Taiwan; 8 Chang Gung Memorial Hospital, 222, Maijin Road, Anle District, Keelung City, 204 Taiwan; 9 National University Hospital, 1E Kent Ridge Road, #13-00, Singapore, Singapore; 10 University of Santo Tomas Hospital, España Boulevard, Sampaloc, Manila Philippines; 11 University of Padjadjaran, JI Pasteur 38, Bandung West, Jawa Barat Indonesia; 12 University of Malaya, Wilayah Persekutuan Kuala Lumpur, 50603 Malaysia; 13 People’s Hospital Peking University Health Sciences Centre, 11 Xizhimen South Street, Western District, Beijing, 100044 China; 14 Tokyo Women’s Medical University, 10-22 Kawada-Cho, Shinjuku, Tokyo 162-0054 Japan; 15 Rheumatology, Tokyo Women’s Medical University, 10-22 Kawada-Cho, Shinjuku, Tokyo 162-0054 Japan; 16 Tan Tock Seng Hospital, 11 Jalan Tan Tock Seng, Singapore, 308433 Singapore; 17 Department of Rheumatology, Flinders Medical Centre, Bedford Park, South Australia 5042 Australia; 18 University of New South Wales and Ingham Institute of Applied Medical Research, 1 Campbell St, Liverpool, New South Wales 2170 Australia; 19 University of Hong Kong, Queen Mary Hospital, 102 Pok Fu Lam Road, Pok Fu Lam, Hong Kong; 20 Janssen Pharmaceutical Companies of Johnson and Johnson, 1125 Trenton Harbourton Rd, Titusville, NJ 08560 USA; 21 School of Public Health, University of Sydney, Sydney, New South Wales 2206 Australia; 22 Department of Rheumatology, Royal Prince Alfred Hospital, Camperdown, NSW 2050 Australia; 23 The University of Melbourne at St Vincent’s Hospital, 41 Victoria Parade, Fitzroy, Victoria 3065 Australia

**Keywords:** Anti-malarials, Autoimmune diseases, Cohort study, Immunosuppressants, Systemic lupus erythematosus, SLEDAI

## Abstract

**Background:**

This study examines the association of standard-of-care systemic lupus erythematosus (SLE) medications with key outcomes such as low disease activity attainment, flares, damage accrual, and steroid-sparing, for which there is current paucity of data.

**Methods:**

The Asia Pacific Lupus Collaboration (APLC) prospectively collects data across numerous sites regarding demographic and disease characteristics, medication use, and lupus outcomes. Using propensity score methods and panel logistic regression models, we determined the association between lupus medications and outcomes.

**Results:**

Among 1707 patients followed over 12,689 visits for a median of 2.19 years, 1332 (78.03%) patients achieved the Lupus Low Disease Activity State (LLDAS), 976 (57.18%) experienced flares, and on most visits patients were taking an anti-malarial (69.86%) or immunosuppressive drug (76.37%). Prednisolone, hydroxychloroquine and azathioprine were utilised with similar frequency across all organ domains; methotrexate for musculoskeletal activity. There were differences in medication utilisation between countries, with hydroxychloroquine less frequently, and calcineurin inhibitors more frequently, used in Japan. More patients taking leflunomide, methotrexate, chloroquine/hydroxychloroquine, azathioprine, and mycophenolate mofetil/mycophenolic acid were taking ≤ 7.5 mg/day of prednisolone (compared to > 7.5 mg/day) suggesting a steroid-sparing effect. Patients taking tacrolimus were more likely (Odds Ratio [95% Confidence Interval] 13.58 [2.23–82.78], *p* = 0.005) to attain LLDAS. Patients taking azathioprine (OR 0.67 [0.53–0.86], *p* = 0.001) and methotrexate (OR 0.68 [0.47–0.98], *p* = 0.038) were less likely to attain LLDAS. Patients taking mycophenolate mofetil were less likely to experience a flare (OR 0.79 [0.64–0.97], *p* = 0.025). None of the drugs was associated with a reduction in damage accrual.

**Conclusions:**

This study suggests a steroid-sparing benefit for most commonly used standard of care immunosuppressants used in SLE treatment, some of which were associated with an increased likelihood of attaining LLDAS, or reduced incidence of flares. It also highlights the unmet need for effective treatments in lupus.

**Supplementary Information:**

The online version contains supplementary material available at 10.1186/s42358-024-00366-y.

## Background

Systemic lupus erythematosus (SLE) is a complex chronic autoimmune disease with myriad manifestations and a predilection for affecting women of childbearing age, presenting with greater severity in people of Asian ethnicity, compared with Caucasians [1]. Although outcomes for lupus patients have improved in the second half of the last century [2, 3], significant morbidity and mortality still exist, and the therapies used that have contributed to these improvements in outcomes are associated with potentially harmful toxic effects. In particular, chronic glucocorticoid use has been shown to be associated with multiple adverse effects, including damage accrual over time [4, 5]. Immunomodulatory or immunosuppressive medications, such as hydroxychloroquine and methotrexate, are often used in an effort to reduce long-term glucocorticoid requirements. Although a steroid-sparing effect has been documented for some of these medications [6, 7], the comparative steroid-sparing effects of these different drugs have not been clearly elucidated. In addition, there is a paucity of data regarding the relative effects of these therapies on various lupus outcomes including disease activity, flares, damage accrual and the attainment of low disease activity states such as the Lupus Low Disease Activity State (LLDAS) [8, 9]. Thus, data on the utility of standard of care SLE medicines is less than that emerging from trials of newer medicines which will join them in the therapeutic armamentarium.

The Asia Pacific Lupus Collaboration (APLC) cohort is one of the largest prospective longitudinal cohorts of SLE patients in the world, with over 2000 patients enrolled from multiple countries across the Asia Pacific Region [10]. In this study, we describe the usage of immunomodulatory medications for SLE in the APLC cohort, their differential associations with corticosteroid use, and through the use of propensity score matching evaluate their relative effects on flares, damage accrual, and attainment of the LLDAS.

## Methods

### Study population

Data from patients enrolled in the Asia Pacific Lupus Collaboration (APLC) cohort were used for analysis [10]. These data were collected prospectively using standardised electronic or paper case report forms across multiple sites and countries. Patient enrolment commenced in November 2013, and data analysis was completed in 2021. Patients in this cohort meet either the 1997 American College of Rheumatology (ACR) Modified Classification Criteria for SLE [11] with at least four of the eleven items present, or fulfil the Systemic Lupus Collaborating Clinics (SLICC) 2012 Classification Criteria [12] with at least four of the seventeen items present, or with lupus nephritis in the presence of at least one immunologic criterion.

### Patient and public involvement

Patients and members of the public were not directly involved in setting the research question or determining the outcome measures. We purposefully reduced the burden of participation on patients by including data collection as a component of routine clinical review. We intend to disseminate published results in a study newsletter suitable for a non-specialist audience. Prior to data collection, eligible participants have provided written informed consent as approved by the Monash University Human Research Ethics Committee.

### Data collection and determination of variables

Data at each patient visit are collected by investigators who are experienced rheumatologists with clinical expertise and research interest in SLE. These data include: patient-reported demographic details from a fixed set of categories at the baseline visit; items pertaining to damage accrual (as defined by the SLICC/ACR (Systemic Lupus International Collaborating Clinics/American College of Rheumatology) Damage Index (SDI)) [13] at baseline and annual visits; data on disease activity (SLE Disease Activity Index [SLEDAI]-2K) [14], flare defined according to the Safety of Estrogens in Lupus Erythematosus National Assessment (SELENA) trial Flare Index (SFI) [15], relevant laboratory tests (anti-dsDNA, C3 and C4, urine analysis, full blood count), Physician Global Assessment (PGA) [16], and medication use at each visit.

The Lupus Low Disease Activity State (LLDAS) is defined as being attained if all of the following criteria are met at any visit: (1) a SLEDAI-2K score ≤4, with no activity in major organ systems (renal, central nervous system, cardiopulmonary, vasculitis and fever); (2) no new features of lupus disease activity compared with the previous assessment; (3) a PGA score ≤ 1; (4) current prednisolone-equivalent dosage ≤ 7.5 mg/day; and (5) standard maintenance dosages of immunosuppressive drugs and approved biologics allowed. However, as the purpose of this study was to evaluate the association of immunosuppressive drugs with outcomes after adjustment for steroid dose, only criteria 1–3 were used to define LLDAS.

### Statistical analyses

For descriptive statistics, mean (standard deviation) or median (25th–75th percentiles) were used. The Chi square test or Fisher’s exact test (where appropriate) were used for categorical variables. For propensity score analysis, a panel (up to 16 visits) propensity score matching technique was used for one drug and one outcome at a time. Variables used for propensity score matching were gender, ethnicity (Asian vs non-Asian), age and disease duration at enrolment (years), SLEDAI activity in each domain (immunologic, nervous system, vasculitis, musculoskeletal, renal, serosal, fever, haematological) defined as present or absent, total SLEDAI-2K score and baseline SLICC/ACR Damage Index (SDI) [13] score. For each drug a panel dataset with matched (1:1) number of drug vs non-drug users was created for each outcome based on propensity scores with a caliper of 0.2. Panel logistic regression models were used to assess the relationship between use of each drug at each visit and being in LLDAS, or experiencing disease flares or damage accrual (≥1-point increase in SDI) at the subsequent visit, with anti-malarial use and prednisolone (or equivalent) dose (categorised as ≤ 7.5 mg/day or > 7.5 mg/day) included as covariates. Data were analysed using STATA version 16.1.

## Results

### Demographic and disease characteristics of the APLC cohort

A total of 1707 patients were studied over 12,689 visits, the majority of whom were female (93.2%) and of Asian ethnicity (87.7%) (Table 1). The median age at SLE diagnosis was 29 (IQR 21–40) years, and median disease duration at recruitment 8 (IQR 4–14) years. Patients had at least one follow-up visit, median 4 (IQR 2–7) visits per individual, over a maximum of 8 years follow-up. Median follow up for patients in this cohort was 2.19 (IQR 1.51–2.99) years and median SLEDAI-2K score at recruitment 4 (IQR 2–6). At the end of follow up 50.83% of patient visits had SDI ≥ 1 as compared to 43.14% at baseline. SLEDAI disease activity was most commonly recorded in the immunologic (83.27% of visits), renal (23.79%), mucocutaneous (10.88%) and musculoskeletal systems (5.55%) ever during follow-up. Other demographic data are presented in Table 1.

**Table 1:** Demographic and visit characteristics of the APLC cohort

	n (%) or median (IQR)
Female	1591 (93.2)
Ethnicity	
Asian	1497 (87.7)
White	172 (10.08)
Other	38 (2.23)
Age at diagnosis, years	29 (21–40)
Age at recruitment, years	40.44 (31.15–50.64)
Disease duration at recruitment, years	8 (4–14)
Disease duration ≤ 2 years at recruitment	305 (17.9)
Follow up, years	2.19 (1.51–2.99)
Total number of visits in the data set	12,689 (100)
Number of visits per individual	4 (2–7)
SLEDAI Score at recruitment	4 (2–6)
SDI at recruitment	0 (0–1)
SDI at the end of follow up	0 (0–1)
Baseline SDI ≥ 1	708 (41.48)
End of follow-up^a^ SDI ≥ 1	6450 (50.83)
On anti-malarial ever during follow-up^a^	8865 (69.86)
On immunosuppressive drugs ever during follow-up^a^	9690 (76.37)
Damage accrued, increase in SDI ≥ 1 ever during follow-up^a^	2095 (16.51)
SLEDAI immunologic activity, number of visits^a^	10,566 (83.27)
SLEDAI nervous system activity, number of visits^a^	38 (0.30)
SLEDAI vasculitis activity, number of visits^a^	105 (0.83)
SLEDAI musculoskeletal activity, number of visits^a^	704 (5.55)
SLEDAI mucocutaneous activity, number of visits^a^	1380 (10.88)
SLEDAI renal activity, number of visits^a^	3019 (23.79)
SLEDAI serosal activity, number of visits^a^	74 (0.58)
SLEDAI fever activity number of visits^a^	39 (0.31)
SLEDAI haematological activity, number of visits^a^	693 (5.46)

*APLC* Asia Pacific lupus collaboration, *CLQ* chloroquine, *HCQ* hydroxychloroquine, *IQR* interquartile range, *SDI* SLICC/ACR damage index, *SLEDAI* SLE disease activity index

SLEDAI immunological activity—includes SLEDAI-2K low complement and increased DNA binding items

SLEDAI nervous system activity—includes SLEDAI-2K seizure, psychosis, organic brain syndrome, visual disturbance, cranial nerve disorder, lupus headache, cerebrovascular accident items

SLEDAI vasculitis activity—includes SLEDAI-2K vasculitis item

SLEDAI mucocutaneous activity—includes SLEDAI-2K rash, and mucosal ulcers items

SLEDAI musculoskeletal activity—includes SLEDAI-2K arthritis and myositis items

SLEDAI renal activity—includes SLEDAI-2K urinary casts, haematuria, proteinuria and pyuria items

SLEDAI serosal activity—includes SLEDAI-2K pleurisy and pericarditis items

SLEDAI fever activity—includes SLEDAI-2K fever item

SLEDAI haematological activity—includes SLEDAI-2K thrombocytopenia and leucopenia items

^a^expressed as percentage of total 12,689 visits

### Usage of standard of care medications in the APLC cohort

#### Frequency of medication use

The majority of patient visits (69.86%) used an anti-malarial agent (either hydroxychloroquine or chloroquine), with a similar number (76.37%) using any immunomodulatory or immunosuppressive drug (methotrexate, sulfasalazine, leflunomide, azathioprine, mycophenolate, cyclosporin, tacrolimus, cyclophosphamide, belimumab or rituximab), at any time during follow-up (Table 1). Excluding anti-malarial and corticosteroid use, the majority of patients had taken either no (47.5%), or one (47.2%) immunosuppressive drug over the course of the follow up period. The most commonly utilised medications in the APLC cohort were prednisolone (82.31% of patients had used it at some point during follow up), hydroxychloroquine (65.08%), mycophenolate mofetil (37.55%) and azathioprine (28.88%), with the least commonly taken medications being leflunomide (0.53%), belimumab (1.82%) and rituximab (2.34%) (Table 2, Supplementary Table 1). The low utilisation of biologicals likely reflects the challenges in accessing biologics for lupus, as these medications are not approved for lupus treatment in much of the Asia-Pacific region. A small percentage of patients had utilised cyclophosphamide (8.03%), chloroquine (7.26%), cyclosporin (5.21%), mycophenolic acid (5.04%), and tacrolimus (3.34%), at any time during the follow up period. There was little variation in the percentage of patients on each medication across visits (Supplementary Table 1).

**Table 2:** Anti-malarials and immunosuppressants usage in the APLC cohort

Medication use during follow up	Yes ever, by patient n = 1707	Yes ever, by visit n = 12,689	Dose in mg/day, median (IQR)
Prednisolone	1405 (82.31)	9960 (78.49)	6.5 (4.88–10)
Hydroxychloroquine	1111 (65.08)	7199 (56.73)	225.74 (200–366.67)
Mycophenolate mofetil	641 (37.55)	2717 (21.41)	1250 (1000–1833.33)
Azathioprine	493 (28.88)	2611 (20.58)	75 (50–100)
Methotrexate	137 (8.03)	782 (6.16)	12.5 (10–17.27)*
Cyclophosphamide	137 (8.03)	397 (3.13)	Not applicable
Chloroquine	124 (7.26)	877 (6.91)	150 (125–150)
Cyclosporin	89 (5.21)	411 (3.24)	107.14 (100–150)
Mycophenolic acid	86 (5.04)	451 (3.55)	952.5 (672.31–1530)
Tacrolimus	57 (3.34)	245 (1.93)	3 (2.5–3)
Rituximab	40 (2.34)	94 (0.74)	Not applicable
Belimumab	31 (1.82)	88 (0.69)	Not applicable
Leflunomide	9 (0.53)	24 (0.19)	20 (20–20)

Results reported as n (%) unless otherwise stated

*APLC* Asia Pacific lupus collaboration, *IQR* interquartile range

*dose in mg/week

#### Medication doses

Median doses of medications taken were generally within standard recommended doses for each medication (Table 2). The median prednisolone dose over the duration of follow up was 6.5 mg/day, for hydroxychloroquine 225.74 mg/day, mycophenolate mofetil 1250 mg/day, mycophenolic acid 952.5 mg/day, azathioprine 75 mg/day, methotrexate 12.5 mg/week, chloroquine 150 mg/day, cyclosporin 107.14 mg/day, tacrolimus 3 mg/day, and leflunomide 20 mg/day.

#### Medication use by disease manifestation

The association of different medications with activity in organ system domains (immunological, central nervous system, vasculitis, musculoskeletal, renal, serositis, fever, haematological and cutaneous) is shown in Table 3. Prednisolone, hydroxychloroquine, and azathioprine were utilized with similar frequency for activity across all organ domains. Mycophenolate mofetil appeared to be less frequently utilized for musculoskeletal (8.95%, expressed as a percentage of visits with a particular organ activity), and central nervous system (10.53%) activity, and fever (5.13%), compared to the other disease manifestations (15.44–36.5%). However, this was not the same for mycophenolic acid, which, although also used infrequently for musculoskeletal manifestations (2.84%), was most frequently utilized for central nervous system activity (13.16%) and fever (15.38%). Methotrexate was used most frequently for musculoskeletal activity (28.27%), compared to other disease manifestations (0–11.16%). Apart from prednisolone and hydroxychloroquine, the most frequently utilised medications for each organ manifestation were: azathioprine (39.47%) and mycophenolic acid (13.16%) for central nervous system activity; mycophenolate mofetil (26.67%) and azathioprine (15.24%) for vasculitis; methotrexate (28.27%) and azathioprine (17.05%) for musculoskeletal activity; mycophenolate mofetil (36.5%) and azathioprine (24.61%) for renal activity; mycophenolate mofetil (20.27%) and azathioprine (18.92%) for serositis; azathioprine (30.01%) and mycophenolate mofetil (15.44%) for haematological activity; and azathioprine (23.22%) and mycophenolate mofetil (19.76%) for cutaneous activity. Cyclophosphamide and rituximab were utilised more frequently for more serious organ involvement (vasculitis (15.24%), renal disease (8.58%) and central nervous system activity (7.89%) for cyclophosphamide; vasculitis (3.81%) for rituximab).

**Table 3:** Medication use by SLEDAI organ manifestation in the APLC cohort

	Imm, n = 10,566	CNS, n = 38	Vasculitis, n = 105	MSK, n = 704	Renal, n = 3019	Serositis, n = 74	Fever, n = 39	Haem, n = 693	Skin, n = 1675
Prednisolone, n = 9960	8319 (83.52) **(78.73)**	36 (0.36) **(94.74)**	98 (0.98) **(93.33)**	582 (5.84) **(82.67)**	2754 (27.65) **(91.22)**	57 (0.57) **(77.03)**	35 (0.35) **(89.74)**	556 (5.58) **(80.23)**	1433 (14.39) **(85.55)**
Hydroxychloroquine, n = 7199	6375 (88.55) **(60.34)**	27 (0.38) **(71.05)**	67 (0.93) **(63.81)**	474 (6.58) **(67.33)**	1514 (21.03) **(50.15)**	60 (0.83) **(81.08)**	27 (0.38) **(69.23)**	518 (7.2) **(74.75)**	1116 (15.5) **(66.63)**
Mycophenolate mofetil, n = 2717	2281 (83.95) **(21.59)**	4 (0.15) **(10.53)**	28 (1.03) **(26.67)**	63 (2.32) **(8.95)**	1102 (40.56) **(36.5)**	15 (0.55) **(20.27)**	2 (0.07) **(5.13)**	107 (3.94) **(15.44)**	331 (12.18) **(19.76)**
Azathioprine, n = 2611	2215 (84.83) **(20.96)**	15 (0.57) **(39.47)**	16 (0.61) **(15.24)**	120 (4.6) **(17.05)**	743 (28.46) **(24.61)**	14 (0.54) **(18.92)**	11 (0.42) **(28.21)**	208 (7.97) **(30.01)**	389 (14.9) **(23.22)**
Methotrexate, n = 782	647 (82.74) **(6.12)**	0 (0) **(0)**	10 (1.28) **(9.52)**	199 (25.45) **(28.27)**	57 (7.29) **(1.89)**	7 (0.9) **(9.46)**	4 (0.51) **(10.26)**	39 (4.99) **(5.63)**	187 (23.91) **(11.16)**
Chloroquine, n = 877	586 (66.82) **(5.55)**	0 (0) **(0)**	5 (0.57) **(4.76)**	60 (6.84) **(8.52)**	113 (12.88) **(3.74)**	2 (0.23) **(2.7)**	4 (0.46) **(10.26)**	32 (3.65) **(4.62)**	185 (21.09) **(11.04)**
Mycophenolic acid, n = 451	418 (92.68) **(3.96)**	5 (1.11) **(13.16)**	0 (0) **(0)**	20 (4.43) **(2.84)**	167 (37.03) **(5.53)**	4 (0.89) **(5.41)**	6 (1.33) **(15.38)**	25 (5.54) **(3.61)**	69 (15.3) **(4.12)**
Cyclosporin, n = 411	348 (84.67) **(3.29)**	1 (0.24) **(2.63)**	1 (0.24) **(0.95)**	13 (3.16) **(1.85)**	189 (45.99) **(6.26)**	6 (1.46) **(8.11)**	1 (0.24) **(2.56)**	37 (9.0) **(5.34)**	55 (13.38) **(3.28)**
Tacrolimus, n = 245	233 (95.1) **(2.21)**	0 (0) **(0)**	3 (1.22) **(2.86)**	11 (4.49) **(1.56)**	40 (16.33) **(1.32)**	1 (0.41) **(1.35)**	0 (0) **(0)**	5 (2.04) **(0.72)**	34 (13.88) **(2.03)**
Leflunomide, n = 24	24 (100) **(0.23)**	0 (0) **(0)**	1 (4.17) **(0.95)**	10 (41.67) **(1.42)**	0 (0) **(0)**	1 (4.17) **(1.35)**	1 (4.17) **(2.56)**	1 (4.17) **(0.14)**	4 (16.67) **(0.24)**
Cyclophosphamide, n = 397	336 (84.63) **(3.18)**	3 (0.76) **(7.89)**	16 (4.03) **(15.24)**	19 (4.79) **(2.7)**	259 (65.24) **(8.58)**	4 (1.01) **(5.41)**	2 (0.5) **(5.13)**	22 (5.54) **(3.17)**	59 (14.86) **(3.52)**
Rituximab, n = 94	93 (98.94) **(0.88)**	0 (0) **(0)**	4 (4.26) **(3.81)**	17 (18.09) **(2.41)**	21 (22.34) **(0.7)**	1 (1.06) **(1.35)**	1 (1.06) **(2.56)**	13 (13.83) **(1.88)**	34 (36.17) **(2.03)**
Belimumab, n = 88	83 (94.32) **(0.79)**	0 (0) **(0)**	0 (0) **(0)**	6 (6.82) **(0.85)**	39 (44.32) **(1.29)**	2 (2.27) **(2.7)**	0 (0) **(0)**	11 (12.5) **(1.59)**	30 (34.09) **(1.79)**

*APLC* Asia Pacific lupus collaboration, *CNS* central nervous system, *Haem* haematological, *Imm* immunological activity, *MSK* musculoskeletal, *SLEDAI* SLE disease activity index

Non-bolded values expressed as n (%) of visits on a particular drug; bolded values expressed as n (%) of visits with a particular organ activity

#### Medication use by country

The frequency of medications ever used during the follow up period across different countries in the APLC cohort is outlined in Table 4 and Supplementary Figure. Prednisolone was used by a majority of patients across countries (62.31–97.94%), as was hydroxychloroquine for the majority of countries, apart from Thailand (27.68%), Indonesia (4.67%) and Japan (3.09%). In Indonesia the frequency of chloroquine use was far higher (70.09%) than in the other countries (13.99% in Thailand, but 0–0.87% for all other countries). Mycophenolate mofetil was more commonly used in Taiwan (63.3%) and Thailand (71.13%); mycophenolic acid most commonly utilised in Australia (13.08%) and Singapore (9.2%). With regard to use of calcineurin inhibitors, there was far higher frequency of tacrolimus use in Japan (43.3%) compared to the other countries (0–3.48%); cyclosporin was also most frequently used in Japan (9.28%) and Thailand (12.2%). The frequency of methotrexate, leflunomide and rituximab use was highest in Australia (15.26%, 1.87%, and 6.54%, respectively). The frequency of cyclophosphamide use was highest in Indonesia (18.69%), Thailand (17.26%) and The Philippines (15.65%); the latter also had the highest frequency of belimumab use (5.22%), alongside Taiwan (5.39%). Azathioprine was used in 24.4–54.21% of patients, apart from in The Philippines (6.96%) and Japan (11.34%).

**Table 4:** Medication use by country in the APLC cohort

	Australia, n = 321	Indonesia, n = 107	Japan, n = 97	Malaysia, n = 184	Philippines, n = 115	Singapore, n = 250	Taiwan, n = 297	Thailand, n = 336
Azathioprine	92 (28.66)	58 (54.21)	11 (11.34)	62 (33.7)	8 (6.96)	90 (36.0)	90 (30.3)	82 (24.4)
Mycophenolate mofetil	70 (21.81)	6 (5.61)	16 (16.49)	10 (5.43)	40 (34.78)	72 (28.8)	188 (63.3)	239 (71.13)
Mycophenolic acid	42 (13.08)	0 (0)	0 (0)	1 (0.54)	1 (0.87)	23 (9.2)	9 (3.03)	10 (2.98)
Methotrexate	49 (15.26)	7 (6.54)	4 (4.12)	5 (2.72)	4 (3.48)	26 (10.4)	22 (7.41)	20 (5.95)
Leflunomide	6 (1.87)	0 (0)	0 (0)	0 (0)	1 (0.87)	2 (0.8)	0 (0)	0 (0)
Cyclosporin	4 (1.25)	5 (4.67)	9 (9.28)	1 (0.54)	0 (0)	22 (8.8)	7 (2.36)	41 (12.2)
Hydroxychloroquine	289 (90.03)	5 (4.67)	3 (3.09)	147 (79.89)	109 (94.78)	235 (94.0)	230 (77.44)	93 (27.68)
Chloroquine	1 (0.31)	75 (70.09)	0 (0)	0 (0)	1 (0.87)	0 (0)	0 (0)	47 (13.99)
Tacrolimus	7 (2.18)	0 (0)	42 (43.3)	0 (0)	4 (3.48)	2 (0.8)	0 (0)	2 (0.6)
Prednisolone	200 (62.31)	104 (97.2)	95 (97.94)	118 (64.13)	102 (88.7)	217 (86.8)	251 (84.51)	318 (94.64)
Rituximab	21 (6.54)	1 (0.93)	0 (0)	5 (2.72)	2 (1.74)	9 (3.6)	2 (0.67)	0 (0)
Belimumab	4 (1.25)	1 (0.93)	0 (0)	1 (0.54)	6 (5.22)	2 (0.8)	16 (5.39)	1 (0.3)
Cyclophosphamide	12 (3.74)	20 (18.69)	4 (4.12)	1 (0.54)	18 (15.65)	15 (6.0)	9 (3.03)	58 (17.26)

Results expressed as n (%) of patients who had ever used medication

*APLC* Asia Pacific lupus collaboration

#### Association of medications with glucocorticoid use

The association of various medications with dosage of prednisolone taken over the follow up period is shown in Table 5. More patients taking anti-malarials (77.13%), immunosuppressants without anti-malarials (66.2%), or both anti-malarials and immunosuppressants (68.15%), were taking ≤ 7.5 mg/day of prednisolone (or equivalent), compared to those not taking these medications, suggesting a steroid-sparing effect of using anti-malarials and immunosuppressants (*p* < 0.001). With regard to the effect of individual drugs, more patients taking leflunomide (87.5%), chloroquine (79.02%), methotrexate (77.88%), hydroxychloroquine (76.9%), azathioprine (71.81%), mycophenolate mofetil (67.4%), and mycophenolic acid (62.75%) were taking ≤ 7.5 mg/day of prednisolone, suggesting that these particular medications may have a steroid-sparing effect, although the differences for leflunomide and methotrexate were not statistically significant.

**Table 5:** Association of anti-malarial and immunosuppressive usage with prednisolone dose

	**Prednisolone dose**		
**Medication**	**≤7.5 mg/day**	**>7.5 mg/day**	*p*
Leflunomide, n = 24	21 (87.5)	3 (12.5)	0.174
Chloroquine, n = 877	693 (79.02)	184 (20.98)	0.014
Methotrexate, n = 782	609 (77.88)	173 (22.12)	0.125
Hydroxychloroquine, n = 7199	5536 (76.9)	1663 (23.1)	<0.001
Azathioprine, n = 2611	1875 (71.81)	736 (28.19)	<0.001
Mycophenolate mofetil, n = 2717	1830 (67.4)	887 (32.65)	<0.001
Mycophenolic acid, n = 451	283 (62.75)	168 (37.25)	<0.001
Cyclosporin, n = 411	199 (48.42)	212 (51.58)	<0.001
Tacrolimus, n = 245	104 (42.45)	141 (57.55)	<0.001
Rituximab, n = 94	37 (39.36)	57 (60.64)	<0.001
Belimumab, n = 88	33 (37.5)	55 (62.5)	<0.001
Cyclophosphamide, n = 397	142 (35.77)	255 (64.23)	<0.001
Anti-malarials (chloroquine or hydroxychloroquine), n = 8072	6226 (77.13)	1846 (22.87)	<0.001
Immunosuppressants (including anti-malarials), n = 7062	4813 (68.15)	2249 (31.85)	<0.001
Immunosuppressants (without anti-malarials), n = 2899	1919 (66.2)	980 (33.8)	<0.001

Results expressed as n (%) of visits where medication was used

#### Association of medication use with flares, LLDAS attainment and damage accrual

The likelihood of attaining LLDAS, having an SLE flare, or accruing damage, when taking an immunosuppressant medication at a particular visit, as compared to those who were not taking that medication, is displayed in Table 6. In this analysis, the likelihood of attaining a particular outcome was determined using a propensity-score-matched logistic regression model, adjusting for prednisolone dose and anti-malarial use. Five outcomes were statistically significantly different. Patients taking azathioprine or methotrexate were less likely to attain LLDAS compared to those who were not (OR 0.67 [0.53–0.86], *p* = 0.001 and OR 0.68 [0.47–0.98], *p* = 0.038, respectively); patients taking tacrolimus were more likely to attain LLDAS compared to those who were not (OR 13.58 [2.23–82.78], *p* = 0.005). Patients taking mycophenolate mofetil were less likely to experience a flare, compared to those who were not (OR 0.79 [0.64–0.97], *p* = 0.025); and patients taking cyclosporin were more likely to experience a flare, compared to those who were not (OR 1.80 [1.04–3.12], *p* = 0.037). None of the drugs studied was associated with a reduction in damage accrual. Notably, the results for mycophenolate mofetil were not mirrored by those for mycophenolic acid, although this may have been influenced by the substantially fewer visits on mycophenolic acid as compared to mycophenolate mofetil. There was no statistically significant difference in attainment of LLDAS, occurrence of any flare or damage accrual for patients on hydroxychloroquine (propensity score adjusted for prednisolone (or equivalent) use and other immunosuppressants) compared to those not.

**Table 6:** Association of medication use with LLDAS, flares and damage accrual—propensity score matched, and adjusted for prednisolone and anti-malarial use

Medication	LLDAS*				Any flare				Damage accrual			
	n	OR	95% CI	*p*	n	OR	95% CI	*p*	n	OR	95% CI	*p*
Azathioprine	4540	**0.67**	**0.53–0.86**	**0.001**	4560	1.18	0.96–1.44	0.116	4560	1.19	0.82–1.71	0.368
Methotrexate	1326	**0.68**	**0.47–0.98**	**0.038**	1326	1.36	0.94–1.97	0.105	1326	0.98	0.51–1.88	0.961
Mycophenolate mofetil	3886	1.12	0.87–1.43	0.381	3896	**0.79**	**0.64–0.97**	**0.025**	3896	1.35	0.87–2.10	0.175
Mycophenolic acid	750	0.80	0.48–1.35	0.410	756	1.42	0.94–2.13	0.094	756	0.46	0.18–1.14	0.093
Leflunomide	20	0.81	0.10–6.54	0.845	12	–	–	–	12	–	–	–
Cyclosporin	706	1.53	0.67–3.48	0.308	712	**1.80**	**1.04–3.12**	**0.037**	472	1.27	0.41–3.94	0.682
Tacrolimus	362	**13.58**	**2.23–82.78**	**0.005**	364	0.46	0.12–1.78	0.261	364	1.48	0.25–8.89	0.666
Belimumab	150	1.33	0.20–8.93	0.769	150	0.86	0.31–2.39	0.770	150	3.35	0.28–39.97	0.340
Rituximab	124	0.47	0.09–2.49	0.370	86	1.42	0.35–5.75	0.624	124	1.63	0.17–15.96	0.677
Cyclophosphamide	676	1.30	0.69–2.43	0.420	684	1.26	0.79–2.01	0.339	684	2.61	0.82–8.34	0.105
Anti-malarials	6880	0.82	0.65–1.03	0.093	6886	0.86	0.71–1.04	0.124	6886	1.26	0.92–1.72	0.155

*LLDAS defined based on criteria 1–3 only

Bold—statistically significant results

*LLDAS* Lupus low disease activity state, *n* number of visits, *OR* odds ratio, *CI* confidence interval, *p* *p *value

## Discussion

This study describes the use of standard of care medications for SLE in a large, multinational prospectively followed cohort, and evaluates associations with glucocorticoid use, likelihood of attaining the LLDAS or experiencing SLE flares and accruing damage. The main findings include significant differences between agents in attainment of LLDAS and glucocorticoid dose, that could influence future therapeutic guidelines for SLE. Whilst the strength of this study is the multinational, multiethnic nature of the cohort, it is limited geographically within the Asia Pacific region, such that the majority of patients had Asian ancestry. We acknowledge that only 97 patients were included from Japan, making any specific inferences in relation to these patients limited.

Anti-malarials have been a mainstay of lupus treatment, with multiple benefits seen in SLE, including decreasing overall disease severity, preventing disease flares [6, 17], increasing survival [17], favourably altering lipid profiles [18] and anti-thrombotic effects [19]. Despite these benefits, only 65.08% of patients in the APLC cohort used hydroxychloroquine, with a further 7.26% using chloroquine, during the observation period. These frequencies are not dissimilar to other lupus cohorts worldwide [20, 21], and may reflect cessation of therapy prior to enrolment in this study due to adverse effects, or the lack of availability in particular countries, for example Japan until 2015 [22, 23].

In this study, immunomodulatory or immunosuppressant use was observed in about half of patients, with the majority of patients taking a single immunosuppressant medication at each timepoint. These frequencies are lower than observed in some clinical trials, but may in part reflect the disease activity levels in this extant cohort. The most commonly utilised immunosuppressants were mycophenolate mofetil and azathioprine, although there were substantial differences between countries, likely reflecting the availability of medications, as well as potential regional differences in practise, such as with calcineurin inhibitors being favoured in Japan. That very few patients were using more than one immunosuppressant at a particular visit enabled the analysis of use compared to non-use of each drug without significant confounding. The use of cyclophosphamide, belimumab and rituximab was overall low, likely a reflection of their ad hoc use for episodes of high or refractory disease activity, and for the biologics, their lack of accessibility in most jurisdictions of the APLC. A limitation of this study was that adherence to prescribed medication was not assessed.

Most patients in the cohort had taken corticosteroids at some point during the follow up period. Our data suggest that anti-malarials and most immunosuppressants were associated with a potential steroid sparing effect, with the majority of patients taking ≤ 7.5 mg/day of prednisolone if also taking leflunomide, chloroquine, methotrexate, hydroxychloroquine, azathioprine, or mycophenolate, compared to those not taking these medications. Despite these apparent effects, the vast majority of patients were using glucocorticoids, consistent with these steroid-sparing effects being incomplete and with unmet need for improved therapies for SLE.

In examining the likelihood of attaining LLDAS, reducing flares and damage accrual with each medication through propensity-scored matched logistic regression, a statistically significant benefit for attaining LLDAS with tacrolimus use, and for mycophenolate in reducing flares, was seen. The generalisability of the benefit seen with tacrolimus should be interpreted in the context of relatively lower numbers of patients having used tacrolimus compared to other medications (n = 57), and of those who did, the majority (73.68%) being Japanese. In contrast, azathioprine and methotrexate use appeared to be less likely associated with LLDAS attainment, and cyclosporin with higher frequency of flare. Although there have been studies showing differences in disease control between two or three concomitant immunosuppressive medications [24], a comprehensive examination of multiple standard of care medications has been little studied previously. Likewise, few studies have examined LLDAS attainment with standard of care medications, although a randomised trial of mycophenolic acid compared to azathioprine showed clear superiority of mycophenolic acid in LLDAS attainment [25]. LLDAS attainment has also been shown to be superior in patients treated with targeted therapies such as anifrolumab [26], belimumab [27], atacicept [28], and baricitinib [29].

No medication was associated with decreased damage accrual, although this may have been difficult to elicit given the relatively low frequency of damage accrual events in the two-year study period and the wide range of individual drugs being analysed. The association of glucocorticoid use with damage accrual in SLE is well-established, and equally well established to be at least in part independent of the association of glucocorticoid use with active disease [4, 30, 31]. In contrast, evidence suggests protective effects of anti-malarial use against damage accrual in SLE [32]. Defining whether immunosuppressants used as standard of care in SLE management actually afford protection against damage accrual is an important research objective in an era when regulators will assess the role of emerging therapies, and potentially the requirement to ‘fail’ such therapies in order to access newer drugs.

## Conclusions

In conclusion, this study provides valuable insights into the use of medications for SLE. The data support a steroid-sparing benefit for most commonly used standard of care immunosuppressants, some of which were additionally found to increase the likelihood of attaining LLDAS, or reduce the likelihood of flares. Although the study has some limitations, in particular the relatively short duration of follow up, our findings should provoke clinicians to examine their treatment practices, and provide a compelling rationale for the development of new therapies that are more likely to yield improved outcomes in SLE.

## Electronic supplementary material

Below is the link to the electronic supplementary material.

Supplementary Material 1

Supplementary Material 2

## Data Availability

The data that support the findings of this study are included within the article or uploaded as supplementary files.

## List of abbreviations

ACR: American college of rheumatology
APLC: Asia Pacific lupus collaboration
IQR: Interquartile range
LLDAS: Lupus low disease activity state
OR: Odds ratio
PGA: Physician global assessment
SDI: SLICC/ACR damage index
SELENA: Safety of estrogens in lupus erythematosus national assessment
SFI: SELENA trial flare index
SLE: Systemic lupus erythematosus
SLEDAI: SLE disease activity index
SLICC: Systemic lupus collaborating clinics
