# Supplementary material for: Association of systemic lupus erythematosus standard of care immunosuppressants with glucocorticoid use and disease outcomes: a multicentre cohort study
Source: Adv Rheumatol. 2024 May 8;64:38. doi: 10.1186/s42358-024-00366-y (PMC13488659; doi:10.1186/s42358-024-00366-y)
Supplement: Supplementary file 1 [file 42358_2024_366_MOESM1_ESM.docx]

**SUPPLEMENTARY FIGURE**

**Medication use by country in the Asia Pacific Lupus Collaboration cohort**

Percentage of patients

|  | Azathioprine | Mycophenolate | Mycophenolic_acid | Methotrexate | Leflunomide | Cyclosporin | Tacrolimus | Rituximab | Belimumab | Cyclophosphamide | Chloroquine | Hydroxychloroquine | Prednisolone |
| --- | --- | --- | --- | --- | --- | --- | --- | --- | --- | --- | --- | --- | --- |
| Australia | 28.6604 | 21.8069 | 13.0841 | 15.2648 | 1.86916 | 5.60748 | 2.18069 | 6.54206 | 1.24611 | 3.73832 | 0.311526 | 90.0312 | 62.3053 |
| Indonesia | 54.2056 | 5.60748 | 0 | 6.54206 | 0 | 0 | 0 | 0.934579 | 0.934579 | 18.6916 | 70.0935 | 4.6729 | 97.1963 |
| Japan | 11.3402 | 16.4948 | 0 | 4.12371 | 0 | 0 | 43.299 | 0 | 0 | 4.12371 | 0 | 3.09278 | 97.9381 |
| Malaysia | 33.6957 | 5.43478 | 0.543478 | 2.71739 | 0 | 0 | 0 | 2.71739 | 0.543478 | 0.543478 | 0 | 79.8913 | 64.1304 |
| Philippines | 6.95652 | 34.7826 | 0.869565 | 3.47826 | 0.869565 | 0.869565 | 3.47826 | 1.73913 | 5.21739 | 15.6522 | 0.869565 | 94.7826 | 88.6957 |
| Singapore | 36 | 28.8 | 9.2 | 10.4 | 0.8 | 2 | 0.8 | 3.6 | 0.8 | 6 | 0 | 94 | 86.8 |
| Taiwan | 30.303 | 63.2997 | 3.0303 | 7.40741 | 0 | 0 | 0 | 0.673401 | 5.38721 | 3.0303 | 0 | 77.4411 | 84.5118 |
| Thailand | 24.4048 | 71.131 | 2.97619 | 5.95238 | 0 | 0 | 0.595238 | 0 | 0.297619 | 17.2619 | 13.9881 | 27.6786 | 94.6429 |
