# Supplementary material for: Association of systemic lupus erythematosus standard of care immunosuppressants with glucocorticoid use and disease outcomes: a multicentre cohort study
Source: Adv Rheumatol. 2024 May 8;64:38. doi: 10.1186/s42358-024-00366-y (PMC13488659; doi:10.1186/s42358-024-00366-y)
Supplement: Supplementary file 2 [file 42358_2024_366_MOESM2_ESM.docx]

**Supplementary Table 1: Frequency of medications used at each visit in the APLC cohort**

|  | **Visit 1,**  **n = 1707** | **Visit 2,**  **n = 1707** | **Visit 3,**  **n = 1654** | **Visit 4,**  **n = 1558** | **Visit 5,**  **n = 1451** | **Visit 6,**  **n = 1020** | **Visit 7,**  **n = 927** | **Visit 8,**  **n = 792** | **Visit 9,**  **n = 510** | **Visit 10,**  **n = 433** | **Visit 11,**  **n = 339** | **Visit 12,**  **n = 239** | **Visit 13,**  **n = 190** | **Visit 14,**  **n = 115** | **Visit 15,**  **n = 43** | **Visit16,**  **n = 4** |
| --- | --- | --- | --- | --- | --- | --- | --- | --- | --- | --- | --- | --- | --- | --- | --- | --- |
| AZA | 362  (21·21) | 374  (21·91) | 355  (21·46) | 330  (21·18) | 307  (21·16) | 212  (20·78) | 192  (20·71) | 157  (19·82) | 107  (20·98) | 82  (18·94) | 62  (18·29) | 36  (15·06) | 18  (9·47) | 9  (7·83) | 6  (13·95) | 2  (50·00) |
| HCQ | 1037  (60·75) | 1026  (60·11) | 982  (59·37) | 941  (60·40) | 865  (59·61) | 594  (58·24) | 533  (57·50) | 437  (55·18) | 257  (50·39) | 210  (48·50) | 159  (46·90) | 84  (35·15) | 46  (24·21) | 18  (15·65) | 10  (23·26) | 0  (0) |
| CLQ | 113  (6·62) | 109  (6·39) | 107  (6·47) | 94  (6·03) | 85  (5·86) | 79  (7·75) | 68  (7·73) | 59  (7·45) | 54  (10·59) | 43  (9·93) | 27  (7·96) | 17  (7·11) | 15  (7·89) | 7  (6·09) | 0  (0) | 0  (0) |
| MTX | 90  (5·27) | 97  (5·68) | 104  (6·29) | 102  (6·55) | 96  (6·62) | 75  (7·35) | 62  (6·69) | 54  (6·82) | 38  (7·45) | 29  (6·70) | 21  (6·19) | 10  (4·18) | 3  (1·58) | 1  (0·87) | 0  (0) | 0  (0) |
| MMF | 237  (13·88) | 256  15·00) | 257  (15·54) | 243  (15·60) | 224  (15·44) | 351  (34·41) | 161  (17·37) | 146  (18·43) | 119  (23·33) | 124  (28·64) | 207  (61·06) | 181  (75·73) | 76  (40·00) | 111  (96·52) | 22  (51·15) | 2  (50·00) |
| MPA | 56  (3.28) | 57  (3.34) | 55  (3.33) | 53  (3.40) | 46  (3.17) | 39  (3.82) | 38  (4.10) | 37  (4.67) | 26  (5.10) | 19  (4.39) | 13  (3.83) | 7  (2.93) | 5  (2.63) | 0  (0) | 0  (0) | 0  (0) |
| LEF | 7  (0·41) | 5  (0·29) | 5  (0·30) | 3  (0·19) | 1  (0·07) | 1  (0·10) | 0  (0) | 0  (0) | 0  (0) | 0  (0) | 1  (0·29) | 1  (0·42) | 0  (0) | 0  (0) | 0  (0) | 0  (0) |
| CYC | 49  (2·87) | 49  (2·87) | 44  (2·66) | 37  (2·37) | 38  (2·62) | 38  (3·73) | 37  (3·99) | 31  (3·91) | 24  (4·71) | 19  (4·39) | 14  (4·13) | 11  (4·60) | 8  (4·21) | 6  (5·22) | 6  (13·95) | 0  (0) |
| TAC | 48  (2·81) | 47  (2·75) | 45  (2·72) | 38  (2·44) | 38  (2·62) | 4  (0·39) | 4  (0·43) | 3  (0·38) | 4  (0·78) | 5  (1·15) | 4  (1·18) | 3  (1·26) | 1  (0·53) | 1  (0·87) | 0  (0) | 0  (0) |
| BEL | 14  (0·82) | 14  (0·82) | 13  (0·79) | 14  (0·90) | 9  (0·62) | 6  (0·59) | 6  (0·65) | 5  (0·63) | 1  (0·20) | 1  (0·23) | 0  (0) | 2  (0·84) | 1  (0·53) | 1  (0·87) | 1  (2·33) | 0  (0) |
| RIT | 11  (0·64) | 15  (0·88) | 7  (0·42) | 10  (0·64) | 6  (0·41) | 4  (0·39) | 4  (0·43) | 4  (0·51) | 6  (1·18) | 10  (2·31) | 8  (2·36) | 6  (2·51) | 3  (1·58) | 0  (0) | 0  (0) | 0  (0) |
| CYP | 72  (4·22) | 60  (3·41) | 50  (3·02) | 43  (2·76) | 36  (2·48) | 32  (3·14) | 26  (2·80) | 21  (2·65) | 14  (2·75) | 12  (2·77) | 10  (2·95) | 7  (2·93) | 7  (3·68) | 5  (4·35) | 2  (4·65) | 0  (0) |
| PNL | 1323  (77.50) | 1311  (76.80) | 1265  (76.48) | 1195  (76.70) | 1107  (76.29) | 800  (78.43) | 741  (79.94) | 634  (80.05) | 423  (82.94) | 359  (82.91) | 284  (83.78) | 204  (85.36) | 165  (86.84) | 104  (90.43) | 41  (95.35) | 4  (100) |

Values expressed as n (%); APLC – Asia Pacific Lupus Collaboration; AZA – azathioprine; BEL – belimumab; CLQ – chloroquine; CYC – cyclophosphamide; CYP – cyclosporin; HCQ – hydroxychloroquine; LEF – leflunomide; MTX – methotrexate; MMF – mycophenolate mofetil; MPA – mycophenolic acid; PNL – prednisolone; RIT – rituximab; TAC – tacrolimus. Bold **n** – total number of visits.
